# Volatile sedation using isoflurane versus intravenous sedation in intensive care unit: a propensity matched case control study

**DOI:** 10.1016/j.aicoj.2026.100118

**Published:** 2026-07-16

**Authors:** Romain Tymen, Kahaia De Longeaux, Pierre Bailly, Erwan L’Her

**Affiliations:** a Medical Intensive Care Unit, CHU Brest, Brest, 29200, France; b LaTIM, Université de Bretagne Occidentale, Brest, 29200, France; c Clinical Data Center, CHU Brest, Brest, 29200, France

**Keywords:** Isoflurane, Volatile sedation, Intensive care unit, Sedation

## Abstract

**Background:**

Sedation in the ICU relies on the use of a combination of intravenous hypnotics and opioids. Volatile anesthetics such as isoflurane or sevoflurane have been explored for sedation in ICU for a few years, based on their pharmacological properties. Even if early studies were promising, recent evidence raised concerns about higher mortality while using sevoflurane in ICU. This study aims to evaluate the impact of volatile sedation with Isoflurane within a real-world ICU setting.

**Methods:**

Within a prospective data warehouse system (ReaSTOC), we screened patients receiving mechanical ventilation for more than 24 hours while being sedated for more than 12 hours. A cohort of 216 patients receiving volatile sedation (isoflurane) was matched with 432 patients sedated using intravenous drugs (midazolam and propofol).

**Results:**

ICU and hospital mortality rates were 40.6% and 43.5% in the volatile sedation group vs. 42.3% and 50.0% in intravenous sedation group (p-value 0.73 and 0.14, respectively). Major adverse events including acute kidney injury and dialysis were similar across both groups. Interestingly, ventilation duration and ICU length of stay were higher in volatile sedation group.

**Conclusion:**

No difference in terms of mortality and adverse events occurrence was observed between intravenous and volatile sedation. These findings support the continued use of volatile sedation (isoflurane) in ICU settings.

## Background

Continuous intravenous sedation with propofol and benzodiazepines is broadly used in ICU [1,2]. These drugs are associated with prolonged accumulation, delayed awakening, hemodynamic instability and delirium occurrence [[1], [2], [3], [4]]. Current international guidelines support protocol-based care, lighter sedation targets, and avoidance of benzodiazepines, yet do not routinely recommend volatile anaesthetics outside the operating room [2,5]. Volatile sedation has emerged as an alternative for critically ill patients, allowing rapid titration, minimal active metabolites, and preserved neurological arousal [[6], [7], [8]].

We conducted a large single-center retrospective study using a comprehensive critical care data warehouse. With a propensity score matching to compare groups, we aimed to evaluate isoflurane sedation adverse events incidence within a heterogeneous population of mechanically ventilated ICU patients.

## Methods

### Data management

Within the framework of the ReaSTOC data warehousing project (ClinicalTrials.gov identifier NCT02893462), physiological and biomedical data from all our medical ICU patients’ stays have been systematically collected since January 1, 2015. Detailed information on ethical approvals, warehouse procedures and clinical trial registration are described in a previous article [9]. We retrospectively screened all ICU stays included in the ReaSTOC dataset using SQL (Structured Query Language) queries and data management tools in Python (Polars 1.32.2, Pandas 2.2.3).

### Patients and settings

Patients admitted between January 1, 2015 and June 1, 2025 were screened. Inclusion criteria were invasive mechanical ventilation (MV) for more than 24 hours, and concomitant sedative drugs usage for more than 12 hours. Exclusion criteria were an age under 18 and intentional medication poisoning.

### Exposure groups

Within our ICU, sedation is either provided using isoflurane (Volatile Sedation: VS) or an intravenous combination of midazolam-sufentanyl / propofol-sufentanyl (Intravenous Sedation: IVS). VS is provided while using a dedicated vaporizer device (Sedaconda®). Sedation is managed on a daily routine according to a protocol-guided approach [10], in which the physician in charge defines a target sedation score value and dosage adjustments are nurse-driven per protocol [11]. The currently used nursedriven protocols are available in the supplementary appendix (A1 A2 A3). Midazolam is over-represented upon propofol for IV sedation. This reflects a unit-specific practice, based on the clinical experience of the ICU team and its perceived favorable balance between sedative efficacy and hemodynamic tolerance in critically ill patients.

### Outcomes

Primary outcome was ICU mortality. Secondary outcomes were hospital mortality, ICU Length Of Stay (LOS), hospital LOS, MV duration, ventilatory free days (VFD), hospital mortality and major adverse events (acute kidney injury, renal replacement therapy (RRT), laboratory values).

### Statistical analysis

We performed a 1:2 propensity score matching. Propensity score was obtained via logistic regression using scikit-learn 1.5 package in python 3.11. Propensity matching was defined a priori, and no other weighting technique was used. No other technique was tested in post-hoc analysis, since it could inflate Type 1 error. We matched 1 patient in the VS group with 2 patients in the IVS group, without replacement, using a caliper method with a threshold set at 0.15.

Propensity score was built using the following criteria: age, sex, BMI, Simplified Acute Physiology Score 2 (SAPS 2), ICU admission diagnosis (balancing the 5 most frequent diagnosis: out of hospital cardiac arrest, acute respiratory failure, acute respiratory distress syndrome, Covid-19 related respiratory failure, septic schock), medical history (hypertension, chronic history of pulmonary disease, blood disease, cancer, diabetes), laboratory values (creatinine level, sodium, pH, pCO_2_ pO_2_, hemoglobin, platelet count). These criteria have been selected upon local potential confounders or previously described confounders [4,6,12].

Covariate balance was assessed by calculating the standardized mean difference (SMD) for each variable before and after matching. An SMD threshold of 0.1 was used to determine adequate balance, as values below this cutoff indicate negligible differences between groups. Since matching was performed using a 1:2 ratio, a weighted SMD was used to assess covariate balance.

After matching, cohort description and statistical analysis (Chi-2 score, MannWhitney Score) were performed using Python 3.11, scikit-learn 1.5 and scipy 1.14 packages.

## Results

### Population before and after matching

**Fig. 1:**
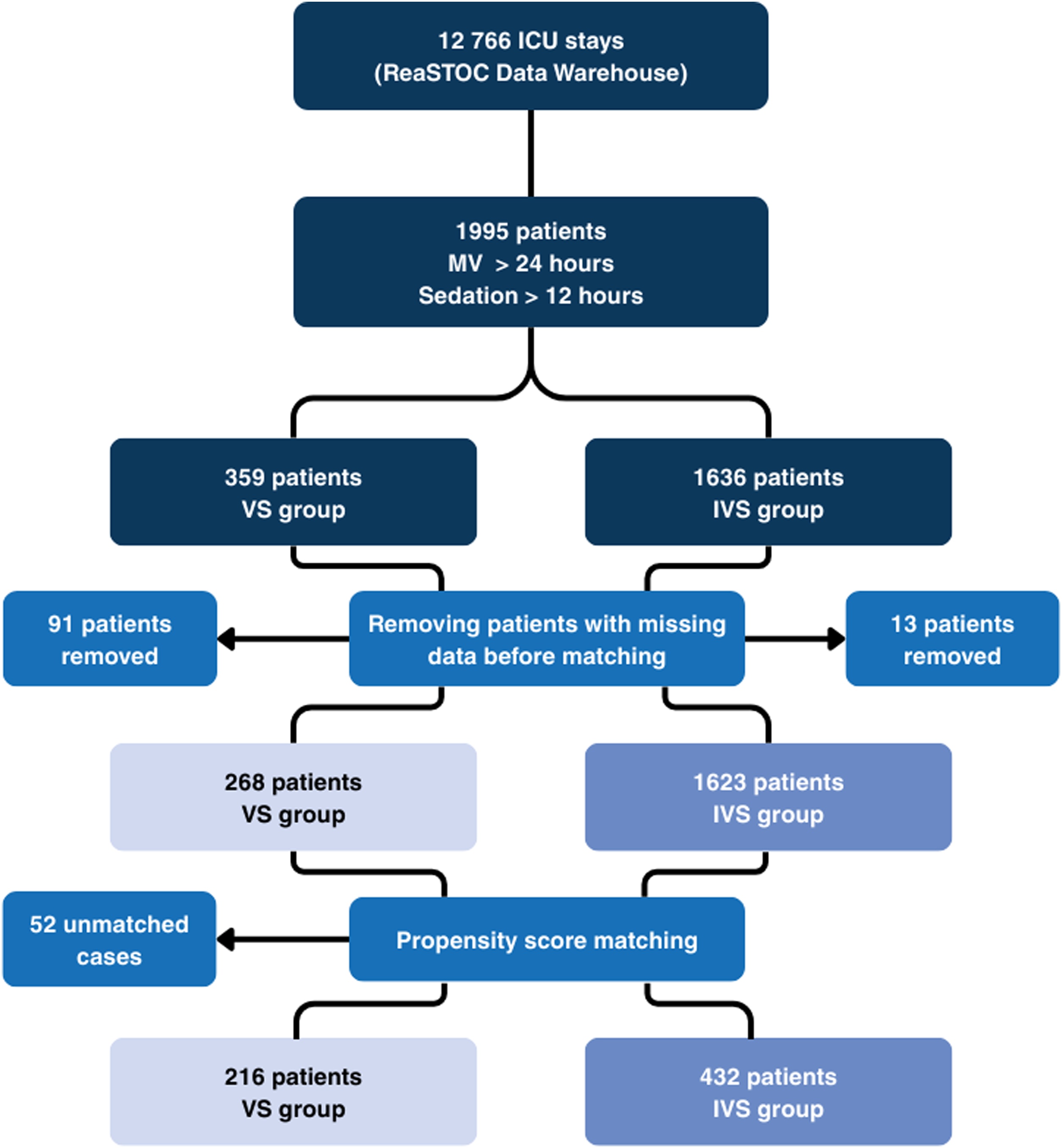
Flowchart of patients’ selection and data processing. VS: Volatile Sedation, IVS: Intravenous Sedation, MV: Mechanical Ventilation.

Among 12.766 ICU stays within the study period, 1995 satisfied inclusion criteria, of whom 359 were sedated using VS. Physiological characteristics of the overall population are provided within Table 1. Flowchart of patients’ selection and data processing is presented within Fig. 1.

**Table 1:** Population baseline characteristics before matching.

Population baseline characteristics before matching			
Feature	VS (n = 268)	IVS (n = 1623)	Overall (n = 1891)
**Physiological parameters**			
Age (years)	59.9 ± 13.6	61.7 ± 14.0	61.4 ± 13.9
Sex (male)	199 (73.8%)	1136 (69.9%)	1335 (70.6%)
SAPS2	55.9 ± 18.8	58.1 ± 18.7	57.7 ± 18.8
Height (cm)	171.0 ± 9.8	169.8 ± 9.5	170.1 ± 9.6
Weight (kg)	80.7 ± 22.4	76.2 ± 20.2	77.0 ± 20.7
BMI (kg/m^2^)	27.6 ± 7.6	26.5 ± 6.7	26.7 ± 6.9
Propofol	–	152 (9.3%)	152 (8.0%)
**Laboratory values**			
pH	7.3 ± 0.1	7.3 ± 0.1	7.3 ± 0.1
pCO_2_ (mmHg)	48.8 ± 14.7	46.8 ± 16.2	47.1 ± 16.0
pO_2_ (mmHg)	93.9 ± 55.6	97.7 ± 61.8	97.0 ± 60.7
Potassium (mmol/L)	4.2 ± 0.7	4.2 ± 0.9	4.2 ± 0.8
Sodium (mmol/L)	138.7 ± 5.0	138.3 ± 6.1	138.3 ± 6.0
Creatinine (μmol/L)	99.6 ± 95.0	121.1 ± 107.3	117.2 ± 105.5
Hemoglobin (g/dL)	12.2 ± 2.7	12.0 ± 2.7	12.0 ± 2.7
Leukocytes (G/L)	20.9 ± 47.3	150.3 ± 2779.5	127.2 ± 2519.4
Platelet count (G/L)	254.0 ± 113.8	226.9 ± 132.5	231.7 ± 129.7
HCO₃^−^ (mmol/L)	23.8 ± 5.6	22.1 ± 6.2	22.4 ± 6.1
**Medical history**			
Hypertension	103 (39.0%)	680 (42.0%)	783 (41.4%)
Diabetes	37 (14.2%)	329 (20.3%)	366 (19.2%)
COPD	45 (17.0%)	255 (15.7%)	300 (15.9%)
Blood malignancy	18 (7.2%)	148 (9.2%)	166 (8.8%)
Solid malignancy	21 (8.4%)	187 (11.6%)	208 (11.0%)
Propensity score	0.341 ± 0.20	0.163 ± 0.14	–

Continuous variables are presented as mean ± standard deviation; categorical variables as count (percentage). Biological variables are the closest value available before sedative drug initiation.

VS: Volatile Sedation, IVS: Intravenous Sedation, BMI: body mass Index, COPD: chronic obstructive pulmonary disease.

All matching criteria had an after matching SMD below 0.1 (Pre and post matching SMD loveplot is available in supplementary appendix A4). An over representation of cardiac arrest in the VS group resulted in 52 unmatched patients. Table 2 summarizes the post-matching balance of all variables, as well as SMD when features were used to perform matching. Table A1 (supplementary appendix) presents the distribution of relevant diagnoses before and after matching.

**Table 2:** Population baseline characteristics after matching.

Feature	VS (n = 246)	Matched IVS (n = 492)	Unmatched IVS (n = 1131)	Overall matched (n = 738)	SMD
**Physiological parameters**					
Age (years)	60.3 ± 13.6	59.9 ± 14.8	60.1 ± 14.0	60.0 ± 14.4	0.031
Sex (male)	176 (71.6%)	351 (71.3%)	785 (69.4%)	528 (71.5%)	0.010
SAPS 2	55.3 ± 19.7	55.7 ± 19.9	55.6 ± 19.8	60.1 ± 19.5	0.016
Height (cm)	170.5 ± 9.7	170.0 ± 9.9	169.0 ± 9.6	170.2 ± 9.8	–
Weight (kg)	80.0 ± 22.2	78.1 ± 21.4	75.3 ± 21.6	78.7 ± 21.7	–
BMI (kg/m²)	27.5 ± 7.3	27.0 ± 7.3	26.6 ± 7.1	27.2 ± 7.3	0.066
Propofol	–	38 (7.7%)	114 (10.0%)	38 (5.1%)	–
**Laboratory values**					
pH	7.3 ± 0.1	7.3 ± 0.1	7.3 ± 0.1	7.3 ± 0.1	0.028
pCO_2_ (mmHg)	48.8 ± 15.0	48.3 ± 16.5	46.2 ± 14.5	48.4 ± 16.0	0.032
pO_2_ (mmHg)	91.4 ± 54.9	93.0 ± 51.8	98.0 ± 53.2	92.5 ± 52.8	0.031
Potassium (mmol/L)	4.2 ± 0.7	4.2 ± 0.8	4.2 ± 0.9	4.2 ± 0.8	–
Sodium (mmol/L)	138.7 ± 4.8	138.9 ± 5.8	138.1 ± 5.2	138.8 ± 5.5	0.043
Creatinine (μmol/L)	107.0 ± 111.9	106.8 ± 74.7	132.1 ± 108.2	106.9 ± 88.8	0.001
Hemoglobin (g/dL)	11.8 ± 2.7	11.8 ± 2.7	12.2 ± 2.6	11.8 ± 2.7	0.002
Leukocytes (G/L)	19.0 ± 29.0	19.6 ± 41.4	160.2 ± 2820.2	19.4 ± 37.7	0.017
Platelet count (G/L)	246.3 ± 109.3	232.3 ± 139.4	215.3 ± 137.5	237.0 ± 130.2	–
HCO₃^−^ (mmol/L)	23.6 ± 5.5	23.1 ± 6.3	21.5 ± 6.2	23.3 ± 6.0	–
**Medical history**					
Hypertension	92 (42.6%)	172 (39.8%)	508 (44.9%)	264 (40.7%)	0.056
Diabetes	35 (16.2%)	71 (16.4%)	258 (22.8%)	106 (16.4%)	0.006
COPD	37 (17.1%)	73 (16.9%)	182 (16.0%)	110 (17.0%)	0.006
Blood malignancy	18 (8.3%)	44 (10.2%)	104 (9.1%)	62 (9.6%)	0.064
Solid malignancy	21 (9.7%)	46 (10.6%)	141 (12.4%)	67 (10.3%)	0.031
Propensity score	0.278 ± 0.17	0.270 ± 0.16	–	0.276 ± 0.15	–

Continuous variables are presented as mean ± standard deviation; categorical variables as count (percentage). All SMD are below 0.1 after matching. SMD was presented only when the variable was used to perform the propensity-score matching. Biological variables are the closest value available before sedative drug initiation.

VS: volatile sedation, IVS: intravenous sedation, SMD: standardized mean difference (presented when feature was used for propensity score matching), BMI: body mass Index, COPD: chronic obstructive pulmonary disease.

### Outcomes

ICU mortality was 40.6% in VS group and 42.3% in IVS group (p value = 0.73) (Table 3).

**Table 3:** Outcomes.

Outcome	VS	IVS	p-value
ICU Mortality (%)	40.6	42.3	0.73
Hospital Mortality (%)	43.5	50.0	0.14
ICU LOS (days)	18.4 (19.4)	13.6 (13.9)	0.002
Hospital LOS (days)	27.2 (33.3)	24.7 (27.1)	0.15
Invasive ventilation duration (days)	15.6 (18.5)	11.6 (16.2)	<0.001
VFD (28 days)	8.7 (10.8)	10.7 (11.4)	0.10
VFD (zeroes)	120 (56%)	208 (49%)	0.03
Norepinephrine consumption (mg)	341 (501)	242 (346)	0.005
Sufentanyl consumption (μg)	600 (787)	297 (452)	0.005

VS: Volatile Sedation group; IVS: Intra-venous Sedation group.

There is no difference in ICU or Hospital mortality between both groups. Total ICU stay is longer in VS group but there is no significative differnce in Hospital LOS. ICU LOS: Intensive care unit lenght of stay, Hospital LOS: hospital length of stay, VFD: ventilatory free days, VFD (zeroes): proportion of zeroes in VFD calculation.

Norepinephrine and sufentanyl consumption are calculated as total given dosis per patient per stay.

In hospital mortality was 43.5% in VS group and 50.0% in IVS group (p-value = 0.14). ICU length of stay, hospital length of stay, invasive ventilation duration, ventilatory free days are presented in Table 3.

AKI incidence is presented in Figure A6 in supplementary appendix. AKI proportion was 29.5% in IVS group and 30.8% in VS group from Day 0 to Day 5 (p-value 0.46). AKI incidence was around 20% in the second and third time windows -day 6 to day 10 and day 11 to day 15- (p-value being respectively 0.90 and 0.89). Renal replacement therapy incidence was 10.1% in VS group and 8.7% in IVS group (p-value = 0.66).

Considering laboratory parameters (Table B2), pH was lower in VS group in the first time window (Day 0–5, p-value of 0.009; Day 6–10, p-value of 0.041). HCO_3_^−^ is higher in the same time windows (p-value of 0.008 and 0.017 respectively). In the first time window, pCO_2_ is higher in VS group (p *<* 0.001). There is no other difference in laboratory parameters distribution between both groups.

## Discussion

In this large propensity score-matched cohort of critically ill patients, volatile sedation using isoflurane was not associated with increased ICU or hospital mortality when compared with intravenous sedation. No difference in any other type of adverse events was observed, which confirms the safety profile of volatile sedation using isoflurane within a real-world ICU population.

Early randomized and observational studies using volatile sedation within the ICU, including recent cardiac arrest cohorts, suggested improved awakening and reduced delirium compared with intravenous regimens [6,13]. In contrast, the SESAR trial, dedicated to patients with acute respiratory distress syndrome (ARDS), reported improved oxygenation but unexpectedly higher 90-day mortality with sevoflurane compared to propofol, driven by a higher incidence of hypotension and acute kidney injury - side effects attributed to the vasodilatory properties and higher fluoride metabolite production of sevoflurane in a population already at high risk of organ failure [12,14,15]. As presented in Table 4, all volatile anaesthetics should not be considered as interchangeable in ICU. These findings are further supported by Jerath et al., who reported comparable safety outcomes with volatile sedation - including isoflurane - in a mixed ICU population [7]. Notably, their 2020 pilot randomized controlled trial specifically evaluated isoflurane for long-term ICU sedation and found it feasible and safe [16]. Meiser et al. demonstrated non-inferiority of inhaled isoflurane versus propofol for ICU sedation in a phase 3 randomized trial, with similar adverse event rates [8]. A subgroup analysis restricted to propofol-sedated patients was not performed, as the proportion of patients receiving propofol within the IVS group was insufficient to yield an adequately powered comparison.

**Table 4:** Potential advantages of isoflurane compared to sevoflurane for volatile-based sedation in the intensive care unit.

Isoflurane vs Sevoflurane Comparison		
**Property**	**Isoflurane**	**Sevoflurane**
Fluoride metabolite production	Low	Higher
Vasodilatory effect	Moderate	More pronounced
Renal safety profile (ICU data)	Favorable (no excess AKI)	Concerns raised (SESAR trial)
Mortality signal in ICU trials	No excess mortality (Meiser et al., present study)	Higher 90-day mortality vs propofol (SESAR trial)
Hepatotoxicity risk	Very low	Very low
Evidence for long-term ICU sedation	Phase 3 RCT (Meiser et al.), pilot RCT (Jerath et al. 2020), observational data	SESAR trial (ARDS), smaller observational studies

AKI: acute kidney injury; ICU: intensive care unit; ARDS: acute respiratory distress syndrome; RCT: randomized controlled trial.

Isoflurane differs pharmacologically from sevoflurane, notably in its lower fluoride metabolite production and more attenuated vasodilatory effect, properties that may underline a more favorable safety profile in a heterogeneous, critically ill population [12,14,15]. The longer duration of mechanical ventilation observed in the isoflurane group is likely attributable to indication bias, patient phenotype, and survivorship effects rather than a direct adverse drug effect, as discussed below.

Isoflurane is preferentially used in our unit for post-cardiac arrest patients, in whom delayed awakening and prolonged ventilation are expected. This indication bias, together with a survivorship effect in the intravenous group (whereby early deaths reduce the apparent accumulation of ventilator days and vasopressor/opioid doses), likely explains the between-group differences in secondary outcomes.

The comparable mortality and organ failure rates suggest that prolonged ventilation under isoflurane more likely reflects treatment strategy and patient phenotype than an adverse effect of volatile sedation itself.

While current international guidelines discourage the routine use of midazolam as a first-line ICU sedative, its continued use in our cohort reflects real-world practice patterns shaped by local protocols, clinician familiarity, and patient-specific considerations - particularly haemodynamic instability, which remains a frequent indication for benzodiazepine-based sedation in critically ill patients. This gap between guideline recommendations and bedside practice is well-documented and reinforces the external validity of real-world observational data. A subgroup analysis restricted to propofol sedated patients was not feasible, as the proportion of patients receiving propofol within the IV sedation group was too limited to yield an adequately powered comparison.

The absence of excess acute kidney injury, renal replacement therapy, or electrolyte disturbances supports that the renal concerns previously observed with sevoflurane – notably in the SESAR trial – do not extend to isoflurane, consistent with its more favorable fluoride metabolite profile [12,14]. The characteristics of unmatched IVS patients are similar to matched IVS patients and therefore supports the generalizability of our findings to the broader IVS population.

Interestingly, some laboratory parameters differed between groups in the first time window of ICU stay (Day 0 to Day 5): lower pH and higher PaCO_2_ and HCO_3_^−^ levels were observed with VS, suggesting a transient respiratory acidosis. This finding is likely related to the use of the Sedaconda® device, which may increase physiological dead space due to the additional volume and resistance of the anaesthetic reflecting filter and the breathing circuit. However, this effect appears to be clinically well tolerated and compensated by renal retention of bicarbonate, consistent with what can be observed with other heat-and-moisture exchange strategies independently of the sedation approach [17].

Several limitations should be acknowledged. First, the single-centre design may affect generalizability. Second, retrospective data collection did not permit recovery of missing values, which may have introduced selection bias. A total of 104 patients were excluded from analysis due to missing data: admission diagnoses were absent in a small number of records, while most exclusions resulted from missing laboratory values occurring when patients admitted from another hospital already had recent blood test results, precluding repeat sampling per our unit’s protocol. Given the limited proportion of missing data, complete-case analysis was preferred over imputation. Even after propensity score matching, unmeasured confounders may persist. Since out of hospital cardiac arrest patients are predominantly sedated with isoflurane in our ICU, this may lead to residual imbalance. The 52 unmatched volatile sedation patients, predominantly post-cardiac arrest, could not be included in the final analysis due to the absence of comparable IVS controls within the pre-defined caliper; generalizability to this specific subgroup should therefore be interpreted with caution. Septic shock remained imbalanced between groups despite propensity score adjustment, likely due to the very limited number of patients with this diagnosis in the volatile sedation group (*n* = 2 after matching). Given the major impact of septic shock on haemodynamic instability and vasopressor requirements, this imbalance should be considered when interpreting between-group differences in norepinephrine consumption. The initial dose of sufentanyl differed between the volatile and intravenous sedation arms, which may reflect differences in baseline clinical characteristics or local sedation protocols, and represents a potential source of residual confounding. Third, sedation depth and delirium - important determinants of clinical outcome - could not be reliably evaluated from retrospective records.

Despite these limitations, the use of a real-world cohort with extensive clinical, physiological and biological data, combined with a careful propensity score matching process, supports the comparability of our groups of interest.

## Conclusion

Isoflurane sedation demonstrated a safety profile compared with intravenous sedation in a real-world propensity matched cohort. This supports its continued use in heterogenous ICU populations and highlight the importance of distinguishing volatile agents: the adverse outcomes reported with sevoflurane in the SESAR trial do not appear to extend to isoflurane. Prospective, multicenter trials stratified by clinical phenotype are warranted to determine whether isoflurane can improve meaningful outcomes such as delirium burden, ventilator-free days, and neurological recovery.

## Take home message

Isoflurane-based volatile sedation is a safe alternative to intravenous sedation in a broad, heterogeneous ICU population, with a pharmacological profile that may confer advantages over sevoflurane. Prospective randomized trials are needed to evaluate patient-specific benefits – including awakening quality, delirium incidence, and ventilation weaning – and to guide individualized sedation strategies.

## Author’s contributions

R.T. had full access to all the data in the study and takes responsibility for the integrity of the data and the accuracy of the data analysis. Acquisition and processing of data: R.T. Statistical analysis and drafting of the manuscript: R.T. Supervision of the study: E.L.H. Drafting and critical revision of the manuscript for important intellectual content: E.L.H, K.D.L, P.B. Final approval of the manuscript: all authors.

## Consent for publication

Not applicable.

## Ethics approval and consent to participate

ReaSTOC (ClinicalTrials.gov identifier NCT02893462) protocol was approved by our local ethics committee (Comité d’ éthique du CHRU de Brest).

## Funding

None.

## Availability of data and material

Not applicable.

## Declaration of competing interest

R.T and K.D.L declares no conflict of interest. E.L.H is the cofounder and shareholder of Oxynov Inc., Canada and Ivanae Medical. He is also consultant for GE Healthcare and Sedana Medical. P.B is investigator coordinator of a study fully funded by Sedana medical.
