# Supplementary material for: Volatile sedation using isoflurane versus intravenous sedation in intensive care unit: a propensity matched case control study
Source: Ann Intensive Care. 2026 Jul 16;16:100118. doi: 10.1016/j.aicoj.2026.100118 (PMC13427550; doi:10.1016/j.aicoj.2026.100118)
Supplement: Supplementary file 1 [file mmc1.docx]

**Appendix A Supplementary materials – propensity score matching**


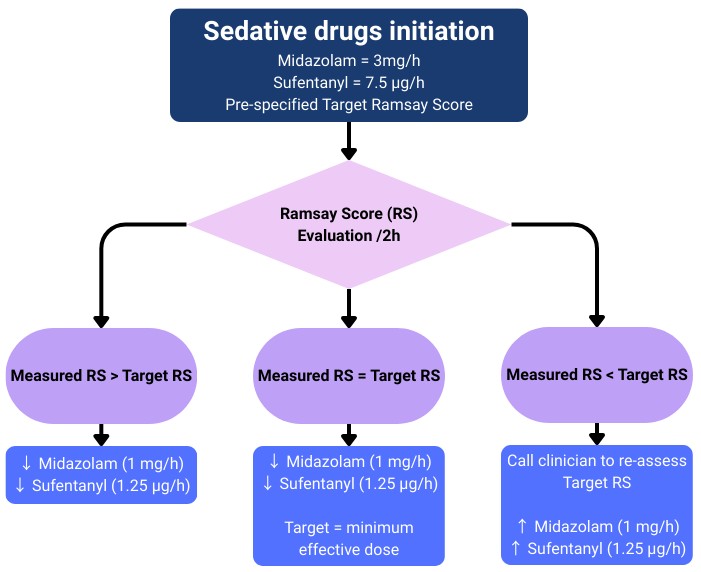


**Fig. A1** Intra-venous midazolam sedation guidelines.

This nurse driven protocole is based on a target Ramsay Score pre-specified by the clinician. This score is re-assessed each 24 hours at least.

Midazolam dilution is 1 mg/mL and Sufentanyl dilution is 2.5 µg/mL, initation dosis are 3 mL/h for each drug.


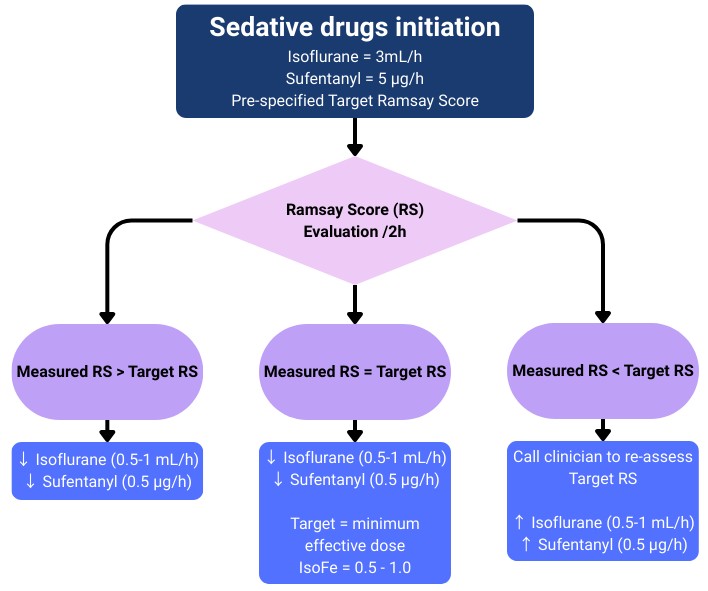


**Fig. A2** Inhaled isoflurane sedation guidelines.

This nurse driven protocole is based on a target Ramsay Score pre-specified by the clinician. This score is re-assessed each 24 hours at least.

Isoflurane is delivered using the Sedaconda® device and Sufentanyl dilution is 2.5 µg/mL.


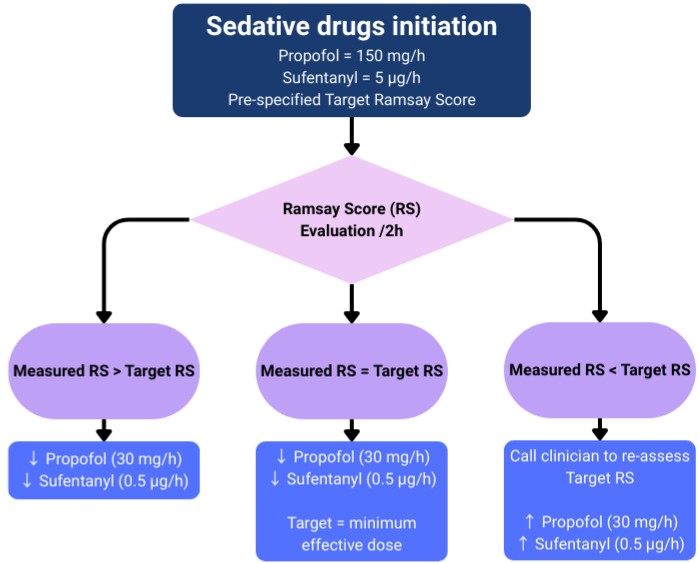


**Fig. A3** Intra-venous propofol sedation guidelines.

This nurse driven protocole is based on a target Ramsay Score pre-specified by the clinician. This score is re-assessed each 24 hours at least.

Propofol concentration is 1 mg/mL and Sufentanyl dilution is 2.5 µg/mL.


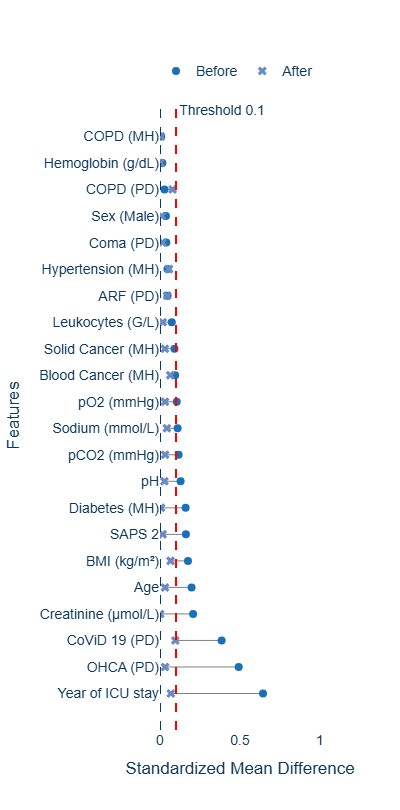


21

**Fig. A4** Loveplot of standardized mean difference (SMD) before and after matching. SMD threshold was set to 0.01 since values below this threshold reflect non-significative difference.

MH: Medical History, PD: principal diagnosis, COPD: chronic obstructive pulmonary disease, ARF:

acute respiratory failure, BMI: body mass index, OHCA: out of hospital cardiac arrest


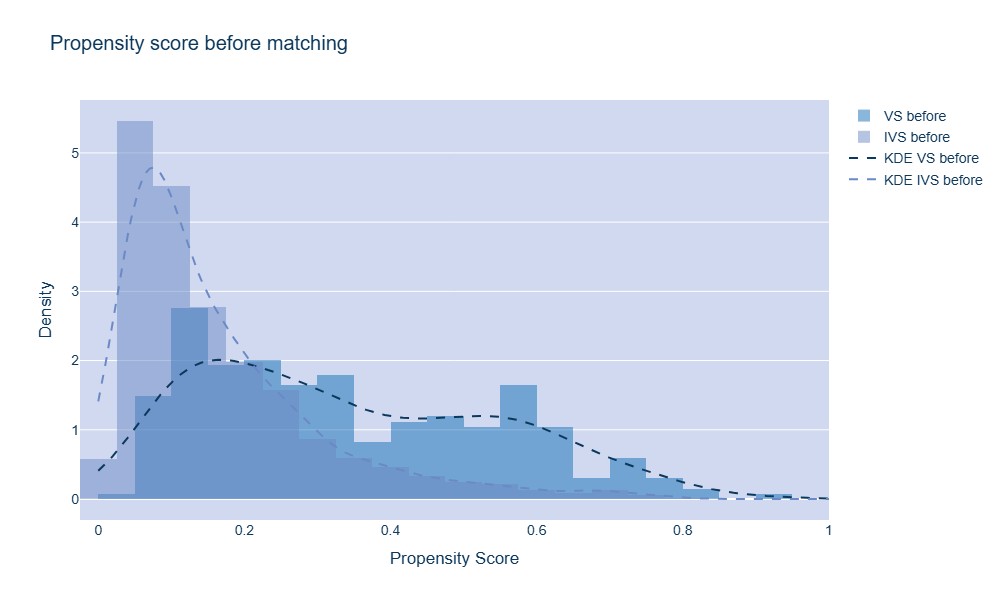


**Fig. A5** Density distribution of propensity scores in the volatile sedation (VS) and intravenous sedation (IVS) groups prior to matching. The propensity score was estimated using logistic regression incorporating age, sex, BMI, SAPS 2, admission diagnosis, medical history, and baseline laboratory values. The limited overlap between groups reflects the imbalance in indication bias, particularly the over-representation of out-of-hospital cardiac arrest in the VS group.


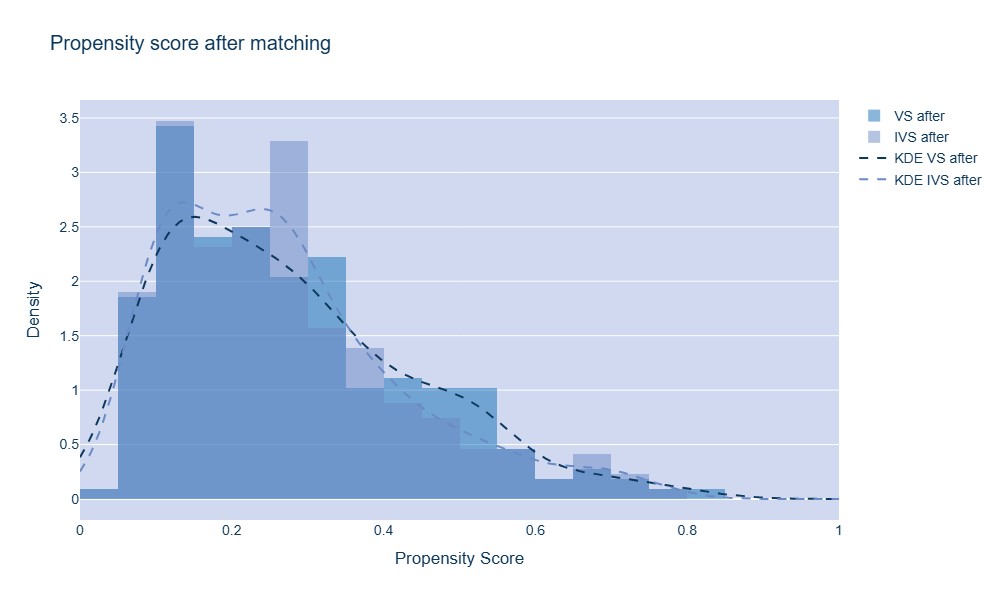


**Fig. A6** Density distribution of propensity scores in the volatile sedation (VS) and intravenous sedation (IVS) groups after 1:2 propensity score matching without replacement (caliper = 0.15). Improved overlap between distributions confirms adequate matching and supports the positivity assumption. All standardized mean differences were below 0.1 after matching.

**Table A1** 5 most frequent diagnosis before and after matching

|  | | **VS** | | **IVS** | |
| --- | --- | --- | --- | --- | --- |
| **Row** | **Diagnosis** | **Count** | **Percentage** | **Count** | **Percentage** |
| **Before matching** | | | | | |
| 1 | OHCA | 102 | 28.4% | 109 | 6.7% |
| 2 | ARF | 17 | 4.7% | 109 | 6.7% |
| 3 | ARDS | 21 | 5.8% | 74 | 4.6% |
| 4 | COVID-19 | 44 | 12.3% | 39 | 2.4% |
| 5 | Septic Shock | 3 | 0.8% | 63 | 3.9% |
| **After matching** | | | | | |
| 1 | OHCA | 36 | 16.7% | 67 | 15.5% |
| 2 | ARDS | 14 | 6.5% | 33 | 7.6% |
| 3 | ARF | 19 | 8.8% | 22 | 5.1% |
| 4 | COVID-19 | 16 | 7.4% | 22 | 5.1% |
| 5 | Septic Shock | 2 | 0.9% | 23 | 5.3% |

VS: Volatile Sedation group; IVS: Intra-venous Sedation group. Diagnosis were identified within the datawarehouse, only principal diagnosis was considered to perform analysis. Principal diagnosis distribution is the same in both groups.

OHCA: out of hospital cardiac arrest, ARF: Acute respiratory failure, ARDS: adult respiratory distress syndrome, COVID-19: CoViD-19 associated respiratory failure.

# Appendix B Supplementary materials - Results


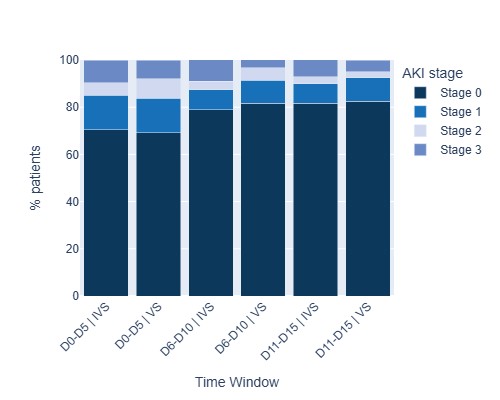


**Fig. B7** Acute kidney injury incidence (AKI) according to sedation group and time windows. Incidence of AKI did not differ between the volatile sedation and intravenous sedation groups across all predefined time windows with Chi² p-value of 0.46 (D1-D5), 0.90 (D6-D10), 0.89 (D11-D15). AKI : Acute kidney injury, VS: volatile sedation, IVS: intra-venous sedation. D0-D5 : Day 0 to Day 5, D6-D10 : Day 6 to Day 10, D11-D15 : Day 11 to 15

**Table B2** Laboratory parameters

| **Parameter** | **Time Period** | **VS** | **n** | **IVS** | **n** | **P-value** |
| --- | --- | --- | --- | --- | --- | --- |
| **Platelet Count (G/L)** | | | | | | |
|  | Day 0-5 | 205 [142-274] | 183 | 179 [103-248] | 375 | **0.03** |
|  | Day 6-10 | 258 [184-342] | 130 | 263 [148-369] | 258 | 0.90 |
|  | Day 11-15 | 306 [232-419] | 109 | 330 [193-475] | 201 | 0.91 |
| **Creatinine (µmol/L)** | | | | | | |
|  | Day 0-5 | 76 [52-156] | 214 | 81 [56-158] | 407 | 0.38 |
|  | Day 6-10 | 65 [46-95] | 152 | 72 [51-116] | 279 | 0.07 |
|  | Day 11-15 | 66 [46-106] | 120 | 66 [49-114] | 235 | 0.49 |
| **Hemoglobin (g/dL)** | | | | | | |
|  | Day 0-5 | 10.1 [8.5-12.1] | 183 | 8.9 [8.4-11.7] | 375 | 0.34 |
|  | Day 6-10 | 9.8 [8.2-11.7] | 131 | 9.4 [8.3-10.9] | 258 | 0.23 |
|  | Day 11-15 | 8.8 [7.9-10.9] | 109 | 9.3 [8.1-11.0] | 201 | 0.62 |
| **Sodium (mmol/L)** | | | | | | |
|  | Day 0-5 | 141 [138-145] | 215 | 140 [138-145] | 410 | 0.31 |
|  | Day 6-10 | 141 [138-147] | 154 | 141 [138-145] | 282 | 0.45 |
|  | Day 11-15 | 140 [137-143] | 124 | 140 [137-143] | 219 | 0.91 |
| **Leukocytes (G/L)** | | | | | | |
|  | Day 0-5 | 12.3 [9.1-15.3] | 183 | 12.0 [8.4-17.8] | 411 | 0.96 |
|  | Day 6-10 | 12.4 [9.1-16.4] | 131 | 12.5 [8.7-18.8] | 257 | 0.64 |
|  | Day 11-15 | 10.9 [7.8-14.4] | 109 | 11.2 [8.2-16.4] | 201 | 0.25 |
| **pCO₂ (mmHg)** | | | | | | |
|  | Day 0-5 | 43 [37-55] | 196 | 39 [33-45] | 364 | **<0.001** |
|  | Day 6-10 | 39 [32-49] | 84 | 39 [34-48] | 145 | 0.72 |
|  | Day 11-15 | 45 [36-52] | 64 | 38 [34-47] | 84 | **0.04** |
| **pO₂ (mmHg)** | | | | | | |
|  | Day 0-5 | 69 [61-79] | 196 | 68 [61-80] | 364 | 0.66 |
|  | Day 6-10 | 66 [57-75] | 84 | 67 [60-80] | 145 | 0.13 |
|  | Day 11-15 | 68 [60-79] | 64 | 68 [60-82] | 84 | 0.63 |
| **Potassium (mmol/L)** | | | | | | |
|  | Day 0-5 | 4.1 [3.6-4.6] | 213 | 3.9 [3.6-4.4] | 461 | 0.07 |
|  | Day 6-10 | 3.9 [3.5-4.2] | 155 | 3.9 [3.6-4.3] | 280 | 0.12 |
|  | Day 11-15 | 4.1 [3.7-4.4] | 124 | 3.9 [3.6-4.3] | 219 | **0.02** |
| **HCO₃⁻ (mmol/L)** | | | | | | |
|  | Day 0-5 | 26 [21-30] | 195 | 24 [21-27] | 364 | **<0.001** |
|  | Day 6-10 | 27 [23-32] | 84 | 25 [22-30] | 144 | **0.04** |
|  | Day 11-15 | 27 [23-33] | 64 | 26 [22-30] | 84 | 0.10 |
| **pH** | | | | | | |
|  | Day 0-5 | 7.38 [7.30-7.43] | 196 | 7.40 [7.32-7.46] | 364 | **0.03** |
|  | Day 6-10 | 7.45 [7.40-7.51] | 84 | 7.43 [7.34-7.48] | 145 | **0.008** |
|  | Day 11-15 | 7.43 [7.37-7.47] | 64 | 7.43 [7.36-7.48] | 84 | 0.54 |

Laboratory parameters were collected upon each period. When there was more than 1 value per patient, mean value was selected to perform overall analysis. pCO_2_ is lower in Day 0-5, as well as a higher HCO_3_^−^ concentration.

VS: Volatile Sedation, IVS: Intra-venous Sedation

Data are presented as follows: median [1^st^ quartile - 3^rd^ quartile]
